# A Synergistic Design Strategy for Gas Storage of Aerogels via Molecular Dynamics Insights into Pore and Surface Chemistry

**DOI:** 10.3390/gels12060509

**Published:** 2026-06-08

**Authors:** Lin Guo, Mu Du, Ying Yin, Gongming Xin

**Affiliations:** 1 School of Nuclear Science, Energy and Power Engineering, Shandong University, Jinan 250100, China; lin.g@sdu.edu.cn; 2 Shenzhen Research Institute of Shandong University, Shenzhen 518057, China; dumu@sdu.edu.cn; 3 Institute for Advanced Technology, Shandong University, Jinan 250100, China; 4 Shandong Key Laboratory of Hydrogen Energy Equipment and Safety, College of New Energy, China University of Petroleum (East China), Qingdao 266580, China; yinyingupc@gmail.com; 5 Shandong Institute of Advanced Technology, Jinan 250100, China

**Keywords:** aerogels, gas adsorption, pore structure, gas–solid interaction, molecular dynamics

## Abstract

The efficient adsorption and storage of gases within nanoporous materials are critical for technologies such as adsorbed natural gas systems and energy storage. A paramount goal is to maximize the adsorbent’s gas uptake capacity. However, the fundamental relationship between pore structure and adsorption performance in disordered aerogels remains unclear, hindering rational material design—specifically, where within the complex pore network adsorption predominantly occurs and how the pore size distribution (PSD) should be engineered to enhance capacity. To address this, we conduct molecular dynamics simulations investigating nitrogen adsorption in silica aerogels with tunable PSDs (achieved via tensile deformation) and varied gas–solid interaction strengths (*ε*). Our results reveal a kinetic-capacity trade-off: microporous-dominated structures saturate rapidly but have limited total uptake, whereas structures with developed mesoporosity (2–10 nm) achieve higher equilibrium capacity via capillary condensation, despite slower kinetics. The interaction strength *ε* is identified as a key factor governing both capacity and selectivity. Synthesizing these insights, we establish dual design guidelines: to maximize storage capacity, a hierarchical network combining micropores and interconnected mesopores is essential; for optimal reversible performance in cyclic applications like adsorbed natural gas, prioritizing open mesopores with moderately tuned surface chemistry is key. This work clarifies key aspects of the structure–performance relationships and provides evidence-based design guidelines for designing advanced aerogel adsorbents tailored for efficient, low-pressure gas storage.

## 1. Introduction

The transition towards sustainable energy systems has intensified the global demand for efficient, safe, and compact technologies for gas storage and separation. Among these, adsorbed natural gas (ANG) for vehicular transportation and low-pressure, ambient-temperature storage of hydrogen or carbon dioxide (CO_2_) represent pivotal applications [1,2]. In particular, CO_2_ capture from industrial flue gas has attracted considerable research attention, with recent studies exploring various porous adsorbents and process configurations for efficient carbon capture [3]. Compared to high-pressure compression or cryogenic liquefaction, adsorption-based storage on porous materials offers notable advantages in energy efficiency and operational safety under milder conditions [4].

Nanoporous aerogels, especially silica-based ones, have emerged as a promising class of materials for this purpose, owing to their ultra-high porosity (typically >90%), extremely low density, and tunable solid matrix [5,6]. Their vast internal surface area (often exceeding 1000 m^2^/g) provides abundant sites for gas molecule adhesion [7]. Yet a persistent gap remains between the theoretical promise and the practically achieved performance of aerogel adsorbents [8]. While it is widely accepted that porosity and surface area are key, the precise microscopic determinants of gas uptake within the inherently disordered pore networks of aerogels remain incompletely understood [9]. This lack of clarity has largely confined material design to empirical trial-and-error rather than rational engineering.

The adsorption behavior is shaped by two coupled factors: geometric confinement from the pore structure and energetic interaction from the surface chemistry [10,11]. On the geometric side, pores are typically classified by size [12,13,14]: micropores (<2 nm) can enhance adsorption through pore filling due to overlapping potential fields from opposite walls; mesopores (2–50 nm) allow capillary condensation, which can dramatically increase storage density; macropores (>50 nm) mainly serve as transport pathways. A central question for random aerogels is which pore size regime contributes most to the equilibrium capacity and what pore size distribution (PSD) is optimal [15,16]. On the energetic side, the surface chemistry—determined by functional groups such as silanols or hydrophobic modifications—dictates the strength of gas–solid interactions [17]. This interaction strength, often represented by the well depth *ε* in molecular models, governs not only adsorption affinity and selectivity but also the enthalpy of adsorption, which directly affects the energy cost for adsorbent regeneration [18,19].

Despite decades of research, the interplay between these two factors remains insufficiently characterized. Experimentally, techniques such as gas physisorption and small-angle X-ray scattering provide essential but ensemble-averaged information; they are unable to dynamically probe molecular-scale adsorption kinetics or disentangle the effects of pore geometry from those of surface chemistry [20,21]. Computationally, most prior simulation studies have relied on idealized pore geometries—infinite slit pores, cylindrical channels, or periodic crystalline frameworks like metal–organic frameworks [22,23,24]. Although a few studies have adopted randomized porous models to examine gas adsorption in aerogel-like structures [25], the specific role of pore size distribution and its interplay with gas–solid interaction strength have not yet been systematically elucidated. In other words, no previous work has independently and simultaneously varied both the PSD and *ε* within a realistic disordered aerogel model to quantitatively decouple their synergistic or antagonistic effects on adsorption capacity, kinetics, and pore-filling mechanisms.

To address this gap, we present a comprehensive molecular dynamics study designed to unravel the adsorption characteristics of gases within silica aerogels featuring random pore structures. Our work pursues three specific objectives: (1) to construct a series of atomistic aerogel models with controlled and systematically varied PSDs via simulated tensile deformation; (2) to independently vary the gas–solid interaction strength (*ε*) to mimic different adsorbates or surface chemistries; and (3) to quantitatively correlate these variables with adsorption capacity, kinetics, and pore-filling mechanisms. This allows us to answer several pivotal questions: For a given interaction strength, where in the random pore network does adsorption predominantly occur—in small pores or large ones? What PSD maximizes equilibrium uptake? And, most importantly, what design principles emerge for the synergistic engineering of pore architecture and surface chemistry to achieve either maximum storage capacity or optimal cyclic reversibility.

The paper is organized as follows. Section 2 presents and discusses the results, starting with model validation, then examining the effects of PSD and gas–solid interaction strength on adsorption behavior. Section 3 summarizes the key findings and proposes design guidelines for advanced aerogel adsorbents in gas storage applications. Section 4 describes the methodology, including the construction of random-pore aerogel models, the simulation setup, and the analysis techniques for quantifying adsorption.

## 2. Results and Discussion

### 2.1. Validation of the Simulation Framework

This study employs molecular dynamics simulations to investigate gas adsorption in amorphous silica aerogels. A series of porous models with tunable pore size distributions were constructed via controlled tensile deformation (10–40 MPa). The gas–solid interaction strength (*ε*) was systematically varied to mimic different contact angle (*φ*): *ε*_1_ = 0.3 (*φ* ≈ 126°), *ε*_2_ = 0.5 (*φ* ≈ 69°), *ε*_3_ = 1.2 (*φ* ≈ 18°), and *ε*_4_ = 1.5 (*φ* ≈ 10°). For reference, *ε* = 1.0 (*φ* ≈ 51°) corresponds to the interaction of real nitrogen with silica. A sandwich-type pressure-controlled simulation system with a force-controlled silica plate was used to maintain constant pressure. Adsorbed molecules were defined using a distance-based cutoff criterion. Detailed simulation procedures are provided in Section 4.

**Figure 1:**
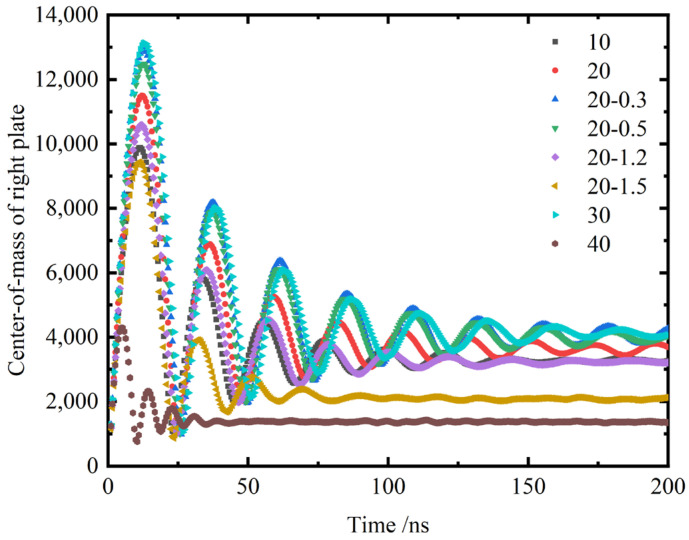
Temporal evolution of the center-of-mass (COM) position of the right, force-controlled silica plate for all simulated conditions. The convergence of all trajectories to a stable plateau within 200 ns confirms the attainment of system-wide mechanical and adsorption equilibrium, validating the simulation protocol.

Figure 1 presents the evidence for system equilibration across all simulation conditions by tracking the temporal evolution of the center-of-mass (COM) position of the right, force-controlled silica plate. As the pressure-control mechanism and the ongoing gas adsorption/desorption processes within the porous aerogel introduce dynamic coupling, achieving a steady state is challenging and requires extended simulation times. The curves in Figure 1 exhibit a consistent trend: an initial transient period characterized by pronounced fluctuations in the COM position, followed by a gradual attenuation of these oscillations. Ultimately, all trajectories converge to a stable plateau where the COM position remains constant within a negligible margin of fluctuation for the remainder of the 200-nanosecond simulation. This convergence to a steady mechanical state indicates that the net force exerted by the confined gas on the plate—which balances the externally applied force (*F*o)—has become invariant with time. This mechanical equilibrium is a direct consequence of the system reaching adsorption equilibrium, where the rates of gas adsorption onto and desorption from the aerogel surface are equal, resulting in a stable number density profile within the pores. The fact that all distinct conditions—spanning different aerogel structures and gas–solid interaction strengths—achieve this plateau within a comparable timeframe validates the sufficiency of the chosen 200 ns simulation duration. Consequently, this timeframe was established as the standard production run length for all subsequent simulations, ensuring that the analyzed adsorption data for all cases are representative of equilibrium conditions, thereby providing a reliable basis for comparative performance analysis.

### 2.2. Defining and Quantifying Adsorption

**Figure 2:**
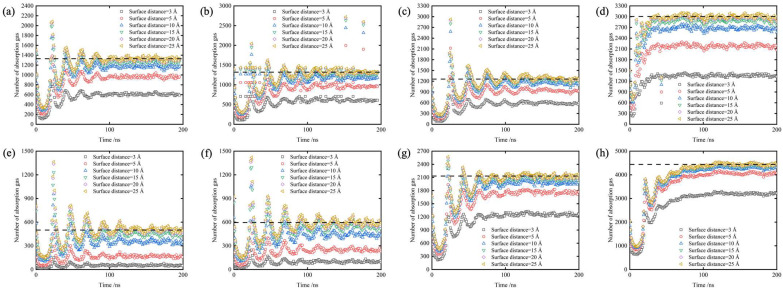
Determination of the critical distance cutoff (*X*) used to define adsorbed gas molecules under various pore sizes ((**a**–**d**): tensile stresses 5–20 MPa at *ε* = 1.0, *φ* ≈ 51°) and contact angles ((**e**–**h**): fixed 20 MPa with *ε* = 0.3/0.5/1.2/1.5 corresponding to *φ* ≈ 126°, 69°, 18°, and 10°, respectively). Since the adsorbed amount changes very little for *X* ≥ 15 Å, so *X* = 25 Å is chosen to provide a small safety margin while ensuring a consistent criterion across all conditions.

A fundamental step in quantifying adsorption from molecular dynamics trajectories is the operational definition of an “adsorbed” molecule. Figure 2 documents the systematic methodology employed in this work to establish a robust and consistent criterion. The number of adsorbed gas molecules was calculated based on the criterion that the shortest distance between a gas molecule and the silica aerogel surface is less than a variable cutoff distance, *X*. To determine an appropriate and physically meaningful value for *X*, we computed the apparent adsorption isotherms for all eight simulated conditions—spanning four pore structures and four distinct gas–solid interaction strengths—using a wide range of *X* values from 5 Å to 25 Å. The resulting adsorption quantities are plotted against *X*.

The trends revealed in Figure 2 are important for interpretation. For small cutoff distances (e.g., *X* < 10 Å), the calculated adsorption number is underestimated across all conditions. This is because such a stringent criterion counts only molecules in the primary, strongly bound adsorption layer. It excludes those in the secondary, more diffuse layers, as well as molecules residing in the centers of small pores where the potential fields from opposite walls overlap. As *X* increases, the computed adsorption amount rises sharply, then gradually transitions into a plateau region for each curve. The emergence of this plateau carries a clear physical meaning: once the cutoff distance becomes sufficiently large to encompass the entire surface-perturbed region—including both the primary adsorption layer and the more diffuse secondary layers—further increasing *X* no longer changes the counted amount, because molecules beyond this distance exhibit bulk-phase density. The convergence of different structures onto a common plateau indicates that the spatial extent of the adsorption-affected zone is insensitive to the specific PSD or interaction strength. We note that for the purely microporous structure, the plateau is reached well before 25 Å, consistent with the expectation that micropore filling is dominated by strong overlapping surface potentials and is essentially complete at smaller distances. A cutoff distance (*X*) of 25 Å, which lies within the common plateau region, was therefore selected as a uniform criterion for this study. While this choice ensures a consistent and basis of comparison across all conditions, the minor systematic overestimation it may introduce for purely microporous architectures should be borne in mind. This systematic approach ensures that subsequent analyses and conclusions regarding adsorption performance are based on a consistent and physically grounded metric.

### 2.3. The Role of Pore Size Distribution in Gas Uptake

**Figure 3:**
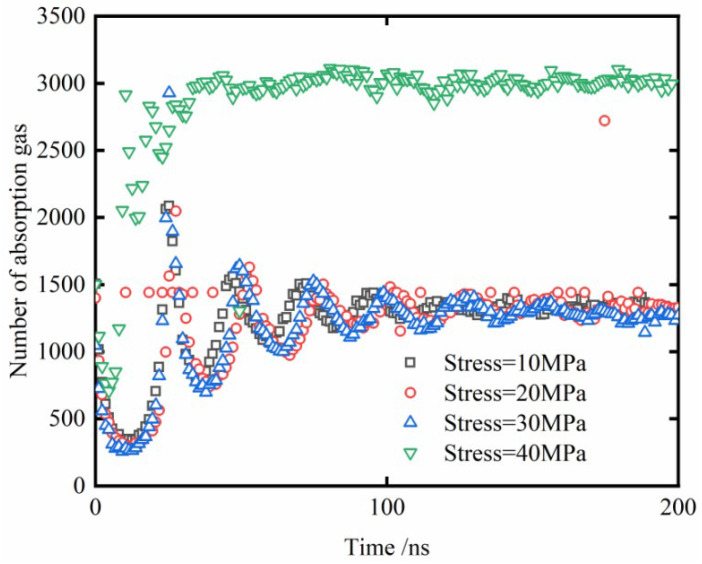
Kinetic profiles of gas adsorption for aerogels with engineered pore size distributions (Structures A–D with stress changing from 10 MPa to 40 MPa). The curves highlight the trade-off between adsorption kinetics and ultimate capacity.

Figure 3 presents the temporal evolution of the adsorbed gas quantity, which serves as the primary metric for evaluating the adsorption performance of the four aerogel structures with engineered PSDs. The kinetic adsorption curves reveal significant and systematic variations in both the adsorption capacity and the rate at which equilibrium is attained, directly linking macroscopic performance to the underlying nano-scale architecture.

A clear hierarchy in equilibrium adsorption capacity is established across the different structures. The aerogel labeled Structure A (corresponding to the lowest tensile strength) exhibits the lowest final adsorbed amount. In contrast, Structure D (subjected to the highest tensile strength) achieves the highest equilibrium adsorption capacity. Structures B and C display intermediate capacities, following a monotonic increase with the degree of tensile deformation applied during their generation. This trend suggests that the adsorption capacity is not governed solely by total porosity but is critically dependent on the specific pore size distribution created by the tensile process. The enhanced capacity in more highly stretched structures likely stems from an optimal combination of increased accessible surface area and a pore network that favors capillary condensation or more efficient packing of adsorbate layers.

Furthermore, the kinetics of the adsorption process are influenced by the pore structure. Structure A, with presumably a denser, more microporous network, shows a rapid initial uptake, saturating within a short simulation time. This indicates fast surface coverage but limited ultimate capacity, characteristic of highly confined pores where adsorption sites are quickly occupied. Conversely, Structure D demonstrates a more gradual adsorption trajectory, taking notably longer to reach equilibrium. This slower kinetics can be attributed to a more open, mesoporous-dominated network. In such a network, gas transport is governed by slower Knudsen or viscous flow diffusion, and filling of larger pores occurs over an extended period. Structures B and C again display intermediate kinetic behaviors.

The more pronounced initial fluctuations observed in the early-stage adsorption curve for the 20 MPa aerogel (Structure B) are noteworthy. These fluctuations can be understood as a signature of its distinctive mesoporous architecture. Unlike the denser, micropore-dominated networks where pore filling proceeds smoothly and continuously, the more developed and broadly distributed mesopores in the 20 MPa structure give rise to transient, non-steady-state filling events—such as the rapid formation and dissipation of local molecular clusters—during the initial uptake period. These dynamic microscopic processes manifest as temporary variations in the instantaneous adsorbed quantity. As the system evolves toward equilibrium, such events become statistically averaged, and the curve converges to a smooth plateau. This behavior confirms that the initial fluctuations represent a physical transient rather than any systematic uncertainty in the simulation.

The divergence in these curves underscores a critical trade-off in aerogel design for gas storage: structures with abundant micropores offer fast filling rates but limited total storage volume, while structures with developed mesoporosity provide higher ultimate capacity at the expense of slower charging times. This finding provides a molecular-level rationale for optimizing adsorbent materials for specific application requirements, such as rapid-cycle buffers versus high-density storage tanks. The results depicted in Figure 3 thus directly address a core research question, demonstrating that “larger pores” within the studied nanoscale range can indeed lead to a higher “total amount adsorbed,” contingent upon allowing sufficient time for the system to reach full equilibrium. These observations are consistent with recent experimental findings on hierarchically porous carbons, where the deliberate introduction of mesoporosity into microporous networks has been shown to enhance low-pressure gas uptake [26].

### 2.4. Tuning Adsorption Performance via Gas–Solid Interactions

**Figure 4:**
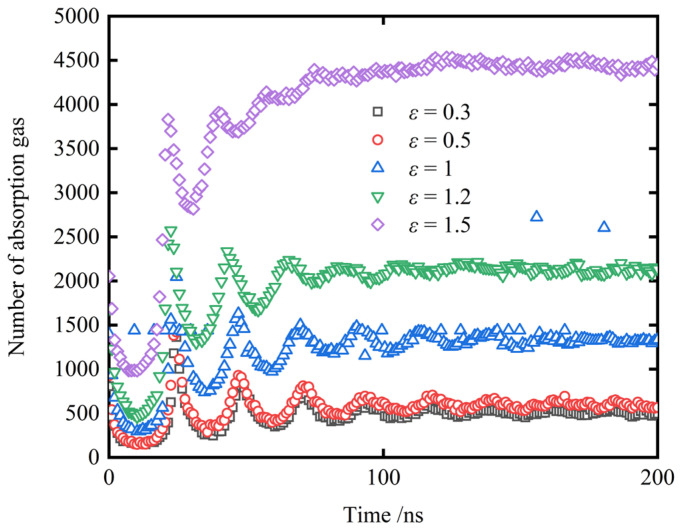
Temporal evolution of gas adsorption on Structure B (stress = 20 MPa) under four gas–solid interaction strengths: *ε*_1_ = 0.3, *ε*_2_ = 0.5, *ε*_3_ = 1.2, and *ε*_4_ = 1.5. The case with *ε* = 1.0 (corresponding to real N_2_–silica interaction) serves as the reference condition.

To systematically isolate the effect of surface chemistry, we selected the 20 MPa structure (Structure B) as the substrate for this parametric study. Owing to its hierarchical network of micropores and mesopores, Structure B is highly responsive to variations in gas–solid interaction strength, whereas in the more stretched 40 MPa aerogel the geometric confinement effects are already approaching saturation. Figure 4 presents the adsorption curves for Structure B (20 MPa) under the four gas–solid interaction strengths varied in this study. The results decouple the effects of surface chemistry from pore geometry, revealing fundamental trends that transcend specific structural details.

A monotonic relationship is observed: as the gas–solid interaction strength (*ε*) increases, both the initial adsorption rate and the final equilibrium adsorption capacity increase substantially. This pronounced sensitivity of adsorption capacity to *ε* is in line with experimental reports on heteroatom-doped porous carbons, where enhanced gas–solid interactions via nitrogen or oxygen functionalization lead to markedly improved uptake at low pressures [27]. The curve corresponding to the weakest interaction (*ε*_1_) exhibits a shallow, slow-rising profile, reaching a low saturation plateau. In contrast, the curve for the strongest interaction (*ε*_4_) displays a steep initial slope, indicative of rapid surface coverage, and ascends to a higher equilibrium adsorbed amount. The intermediate cases (*ε*_2_, *ε*_3_) follow this progression in an orderly manner. This trend is physically intuitive: a stronger attractive potential lowers the energy barrier for adsorption, enhancing the sticking probability of incident gas molecules and increasing the average residence time on the surface. This not only accelerates the filling of available sites but also promotes the formation of denser, more stable adsorbed layers, ultimately leading to a higher total uptake.

The implications of this parametric study extend beyond a single gas type. By systematically varying *ε*, the simulation captures the trend of how adsorption behavior varies with gas–solid interaction strength, providing a qualitative physical picture for how different gas species would behave on the same porous scaffold. For instance, a larger *ε* can represent a gas with greater polarizability or permanent dipole moment (e.g., CO_2_) interacting with a polar silica surface, while a smaller *ε* may model a more inert gas like CH_4_ or N_2_. Therefore, Figure 4 suggests the potential for adsorption selectivity of the given aerogel structure. The significant vertical separation between the curves at equilibrium suggests that a material with a finely tuned surface chemistry could be tailored to discriminate between gases based on their interaction strength, providing a physical basis for guiding the design of separation membranes or selective adsorbents.

### 2.5. Structural Transformation: Pore Evolution Induced by Adsorption

**Figure 5:**
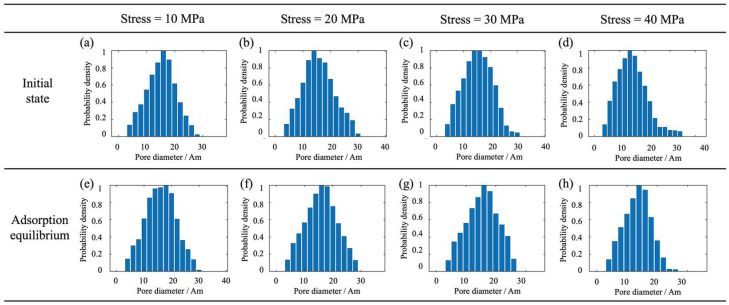
Pore size distributions (PSDs) of the four aerogel models under varying tensile stresses (5, 10, 15, and 20 MPa). For each stress condition, the upper row (**a**–**d**) shows the initial state before adsorption, and the lower row (**e**–**h**) shows the state after adsorption equilibrium. All cases were simulated with *ε* = 1.0.

**Figure 6:**
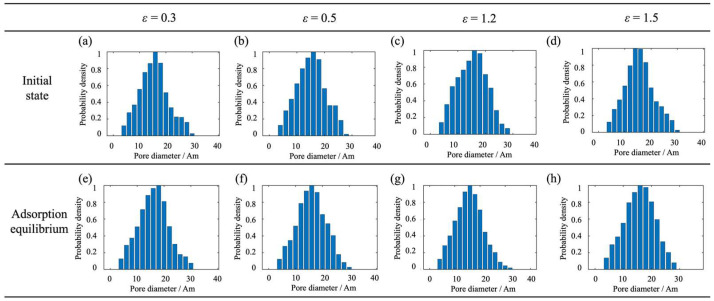
Pore size distributions (PSDs) of the aerogel model (Structure B, 20 MPa) under different gas–solid interaction strengths (*ε* = 0.3, 0.5, 1.2, and 1.5). For each *ε* value, the upper row (**a**–**d**) shows the initial state, and the lower row (**e**–**h**) shows the state after adsorption equilibrium.

Figure 5 and Figure 6 provide a direct structural explanation for the divergent adsorption behaviors observed in Section 2.3. They compare the pore size distributions (PSDs) of the four aerogel models in their initial state and after reaching adsorption equilibrium. Figure 5 presents the PSDs under varying tensile stresses (5, 10, 15, and 20 MPa) for both the unloaded and equilibrated states. The initial PSDs (represented by solid lines) reveal a systematic evolution: Structure A exhibits a narrow distribution concentrated in the micropore range (<2 nm). With increasing tensile strain (Structures B to D), the distributions progressively broaden and shift toward larger diameters, developing substantial tails extending into the mesopore regime (2–50 nm). Structure D, in particular, displays a broad, quasi-bimodal distribution, indicating a highly heterogeneous network rich in mesopores.

The PSDs after adsorption equilibrium (dashed lines) demonstrate a profound and consistent transformation across all structures: pores below approximately 1.0–1.5 nm are largely eliminated. For Structures A and B, this results in a sharp truncation of the microporous portion of the distribution. For Structures C and D, the entire PSD curve shifts uniformly toward smaller diameters. This shift is largest for Structure D, where the broad mesopore peak is attenuated and displaced leftward, indicating a substantial reduction in effective pore volume across the mesopore range.

Figure 6 complements this analysis by showing the PSD evolution under different wettability parameters (simulated via varying gas–solid interaction strength *ε*) while maintaining a fixed pore structure (e.g., Structure B at 20 MPa stress). A trend emerges: as the interaction strength *ε* increases, the post-adsorption PSD (dashed lines) shifts to a greater extent toward smaller diameters compared to the initial state (solid lines). This indicates that stronger gas–solid interactions lead to thicker adsorbed films or more extensive capillary condensation, thereby reducing the free pore radius to a greater degree.

The comparative analysis of Figure 5 and Figure 6 elucidates the dominant adsorption mechanisms. The complete filling of the smallest micropores (below ~1.5 nm) signifies volume filling as the primary mechanism in micropores, where the overlapping potential from opposite walls results in swift and complete saturation. The uniform leftward shift of the mesopore PSDs, especially under higher *ε*, is characteristic of multilayer adsorption and capillary condensation. Here, adsorbate layers line the pore walls, effectively reducing the accessible pore volume. The magnitude of this shift correlates directly with the final adsorption capacity shown in Figure 3: Structure D, with the largest initial mesopore volume, undergoes the greatest PSD modification and achieves the highest gas uptake. This confirms that the adsorbed phase constitutes a significant fraction of the total pore volume, particularly in materials with developed mesoporosity.

Furthermore, the sensitivity of the PSD shift to *ε* (Figure 6) highlights the synergistic role of surface chemistry. Stronger interactions not only enhance monolayer coverage but also promote the growth of multilayers and the onset of condensation at lower relative pressures, thereby amplifying the pore-filling effect in mesopores.

Consequently, the key design principle emerging from this analysis is that maximizing gas storage capacity calls for an architecture that provides a substantial volume of pores within a size range (e.g., 2–10 nm) that is conducive to capillary condensation under the operating conditions. This should be coupled with a surface chemistry tailored to strengthen the gas–solid interaction, thereby enhancing the packing density within the pores. This integrated approach moves beyond merely maximizing ultra-micropores and instead utilizes the synergistic potential of hierarchical porosity and tuned surface energetics.

## 3. Conclusions

In this study, the synergistic roles of pore architecture and surface chemistry in governing gas adsorption within silica aerogels are elucidated through molecular dynamics simulations. The following key conclusions can be drawn.

The adsorption performance is shown to be governed by an interplay between geometric confinement and interfacial energetics. While rapid initial uptake is enabled by micropores via volume filling, high equilibrium capacity is achieved primarily through capillary condensation within interconnected mesopores (2–10 nm), albeit with slower adsorption kinetics. This illustrates a fundamental trade-off between adsorption rate and ultimate storage capacity. The gas–solid interaction strength (*ε*) is identified as a decisive parameter, governing both adsorption capacity and selectivity. Enhanced interaction strengths are shown to improve low-pressure uptake, although they may also hinder desorption in cyclic processes—a trade-off that warrants consideration in practical design.

From these insights, a dual-pathway design framework can be proposed. For the maximization of storage capacity, a hierarchically porous network integrating mesopores as primary reservoirs with tailored micropores as high-affinity sites is recommended, combined with surface functionalization to strengthen gas–solid interactions. For applications requiring high reversibility, such as pressure-swing adsorption, emphasis should be placed on open mesopores with moderately tuned surface affinity to balance working capacity with efficient regeneration.

In summary, these findings suggest that the optimization of aerogel-based adsorbents benefits from a coordinated strategy in which pore structure and surface chemistry are engineered concurrently. It should be noted that the present simulations treat the aerogel framework as rigid, which neglects potential matrix swelling or local structural relaxation that may occur under high pressure or strong gas–solid interactions. While this simplification is justified for isolating the primary adsorption mechanisms, capturing such coupled mechanical effects would be a valuable direction for future work. It should also be noted that the coarse-grained N_2_ model adopted in this study does not explicitly capture polarization, quadrupole moment, or quantum effects. For gases with stronger specific interactions, these factors may modestly influence the quantitative details. We therefore regard the present findings as primarily informative for non-polar or weakly polar adsorbates. Future work should focus on the experimental validation of these design concepts, with simulations extended to flexible frameworks and multi-component gas mixtures to better approximate real-world conditions.

## 4. Materials and Methods

### 4.1. Model Construction

**Figure 7:**
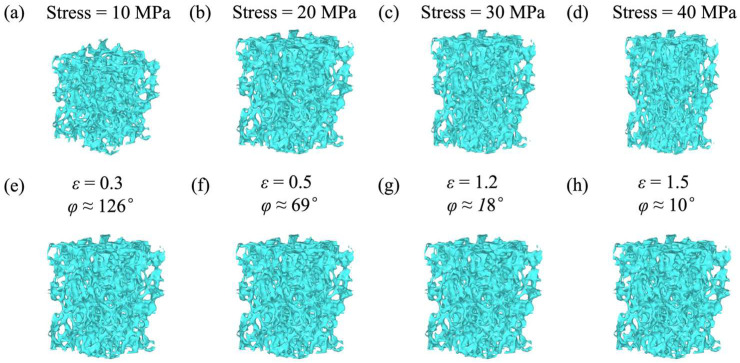
Illustration of the silica aerogel models with tunable pore structures and surface chemistries. (**a**–**d**) Models subjected to varying tensile stresses of 5, 10, 15, and 20 MPa, all at a fixed gas–solid interaction strength of *ε* = 1.0 (corresponding to a water contact angle *φ* ≈ 51° for real N_2_–silica interactions). (**e**–**h**) The 20 MPa model under four different *ε* values: 0.3 (*φ* ≈ 126°, hydrophobic), 0.5 (*φ* ≈ 69°, hydrophilic), 1.2 (*φ* ≈ 18°, strongly hydrophilic), and 1.5 (*φ* ≈ 10°, nearly superhydrophilic).

Figure 7 schematically illustrates the core strategy for fabricating model aerogels with tunable pore structures in this molecular dynamics study. The amorphous silica aerogel was prepared using a melt-quench method from full-density crystal silica, including initialization, heating to an extremely high temperature, and then quenching to ensure sufficient amorphicity. The silica is described by the Tersoff potential parameterized for Si–O systems [28]. The obtained radial distribution function and relevant bond parameters in the amorphous structure agree with the data in the literature [29]. Details of the preparation procedure can be found in our previous work [30]. To systematically investigate the influence of pore geometry on gas adsorption, a pristine aerogel matrix was subjected to four different tensile strengths (i.e., 10, 20, 30, and 40 MPa) [31]. After energy minimization and equilibration, this process yielded four distinct aerogel configurations, visually depicted in the figure, which exhibit a progressive evolution in pore size and spatial disorder with increasing tensile load. This approach successfully generates a series of structures with controlled variations in porosity and pore size distribution (PSD), decoupling the structural variable from material composition.

Furthermore, to isolate and examine the effect of gas–solid interfacial energetics, the 20 MPa-stretched structure was selected as a representative scaffold. The gas–solid interaction strength is governed by the Lennard-Jones well depth *ε*. Based on our previous work [32], the relationship between *ε* and the water contact angle *φ* is given by:

*φ* = 156.9 exp(−1.16(*ε* − 0.01)^2^). (1)

A value of *ε* = 1.0 (*φ* ≈ 51°) represents the interaction of real nitrogen with the silica surface and was used throughout the pore-structure study (Section 2.3 and Section 2.5). To systematically explore the role of surface chemistry, four additional *ε* values were adopted in Section 2.4: *ε* = 0.3 (*φ* ≈ 126°, hydrophobic), 0.5 (*φ* ≈ 69°, hydrophilic), 1.2 (*φ* ≈ 18°, strongly hydrophilic), and 1.5 (*φ* ≈ 10°, nearly superhydrophilic). This dual-variable design forms the foundational basis for all subsequent simulations, enabling a comprehensive analysis of how adsorption behavior is governed by the synergistic or competing effects of physical confinement and interfacial forces. The coarse-grain nitrogen model is used to represent the gas molecules. The interactions for silica are described by Tersoff potential [33]. The interactions between nitrogen-silica are also described by Lennard-Jones potential.

### 4.2. Simulation Protocol

**Figure 8:**
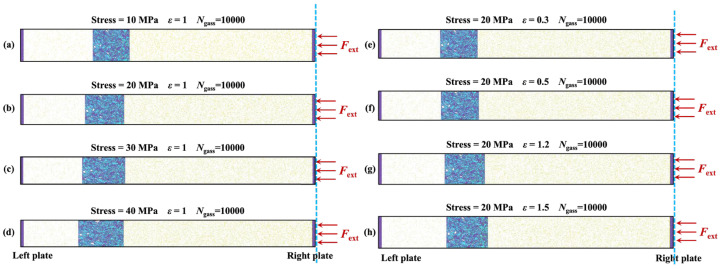
Schematic diagram of the molecular dynamics simulation setup. The system features a sandwich configuration with the silica aerogel (held rigid) confined between two silica plates. Pressure is dynamically controlled by applying an external force to the right plate, while the left plate serves as a fixed boundary. (**a**–**d**): tensile stresses 5–20 MPa at *ε* = 1.0, *φ* ≈ 51°; (**e**–**h**): fixed 20 MPa with *ε* = 0.3/0.5/1.2/1.5 corresponding to *φ* ≈ 126°, 69°, 18°, and 10°, respectively.

Figure 8 depicts the detailed simulation setup employed in this study. A sandwich configuration was constructed, consisting of two rigid silica plates flanking a central silica aerogel composite, with a prescribed number of coarse-grained nitrogen molecules randomly distributed within the accessible pore space to serve as the adsorbate medium. To enhance computational efficiency while focusing on the adsorption dynamics, the aerogel structure was held rigid and fixed in its initial position, a simplification given that the silica–gas interactions are the primary determinant of the adsorption/desorption processes. The left silica plate serves as a fixed physical wall, establishing a boundary condition to prevent gas molecules from permeating beyond the simulation domain. The system pressure is dynamically controlled by applying an external force to the right silica plate. The relationship between the applied force per atom (*F*o) and the target pressure (*p*) is defined as:

*p* = *F*o*N*s/S, (2)

where *N*s is the total number of atoms in the right plate and *S* is its cross-sectional area. For instance, to maintain a pressure of 1 atm, *F*o is set to 4.81 × 10^−7^ eV∕Å. In this setup, the external force *F*o is fixed at a value corresponding to the desired pressure. As gas molecules adsorb onto the aerogel, the pressure they exert on the right plate changes; the plate accordingly adjusts its position until the force from the confined gas balances Fo, thereby maintaining the system at the target pressure. The convergence of the plate position to a stable plateau (Figure 1) confirms that this mechanical equilibrium is reached, ensuring that all adsorption data are collected under steady pressure conditions. To ensure that the pressure-control device itself does not contribute to the measured adsorption, the right plate interacts with gas molecules solely through a purely repulsive Lennard-Jones potential, functioning as an inert piston; molecules in its immediate vicinity were excluded from the adsorption analysis. This controlled setup effectively isolates the phenomena of interest, enabling the precise investigation of gas adsorption under defined confinement and pressure conditions. All simulations are performed at 300 K and 1 atm using the NVT ensemble with a timestep of 0.01 ps. To ensure statistical reliability and capture the complete adsorption equilibrium, our study employs large-scale systems (on the order of 10^5^ atoms) and extended production runs of 200 nanoseconds for each simulated condition.

### 4.3. Data Analysis and Adsorption Quantification

The number of adsorbed gas molecules was determined using a distance-based criterion. A molecule is considered adsorbed if the shortest distance between it and the surface atoms of the silica aerogel is less than a critical cutoff distance, *X*. The rationale and systematic determination of an appropriate *X* value (25 Å) are detailed in Section 2.2, where results computed with various *X* are analyzed to determine a physically justified threshold.

Adsorption equilibrium was considered achieved when the adsorbed amount, calculated using the aforementioned criterion, reached a steady state. This was assessed by monitoring the temporal evolution of the adsorbed gas number; a plateau in this quantity, with fluctuations within a negligible margin over a prolonged simulation time (e.g., the final 50 ns of a 200 ns run), indicated that the net rates of adsorption and desorption were equal.

The pore structure of the aerogel models, both before gas introduction and after reaching adsorption equilibrium, was characterized. The PSD was calculated using a geometry-based algorithm applied to the atomic coordinates of the silica framework, which were explicated in our published work [34]. This allowed for direct comparison of how the effective pore geometry evolved due to gas uptake.

## Data Availability

The original contributions presented in this study are included in the article. Further inquiries can be directed to the corresponding author.

## References

[1] Dunikov D.O., Bezdudny A.V., Blinov D.V., Eronin A.A., Kazakov A.N., Romanov I.A., Ishkov A.G., Romanov K.V., Koloshkin E.A. (2025). Extraction of natural hydrogen from a natural gas probe by LaNi_4.8_ A_l0.2_ metal hydride. Int. J. Hydrogen Energy.

[2] Shinkevich P.S., Velmozhina K.A., Politaeva N.A., Chusov A.N. (2026). Assessment of the ability of microalgae to absorb carbon dioxide in various conditions using Chlorella kessleri and other species. Int. J. Hydrogen Energy.

[3] Rosa D., Segneri V., Di Palma L., Vilardi G. (2023). Synthesis and CO_2_ adsorption capacity of biomass waste functionalized by nanoparticles. Chem. Eng. Trans..

[4] Ng M.S., Wang G., Chakraborty A., Saha B.B. (2025). Highly efficient hydrogen storage on porous materials under cryo-adsorption conditions. Next Energy.

[5] Li Z.J., Liu J.Y., Yu Y., Chang K.J., Wang H., Li Y.J., Gai K. (2022). Rational Design of High-Performance Cationic Organic Network Adsorbents. ACS Appl. Mater. Interfaces.

[6] Gu P., Liu X., Hu Z., Sun Z., Lu L., Yang X., Zu G., Huang J. (2025). Stretchable and Recyclable Nanoporous Semiconducting Polymer Aerogels for Highly Sensitive Biosensors, Electronic Skins, and Artificial Synapses. Adv. Funct. Mater..

[7] Guo L., Yang M., Wang J., Lu Y., Li Z., Zhang S., Xiang G. (2025). Pressure and humidity effects on water adsorption and thermal conductivity of nanoporous aerogels in spacecraft thermal protection system. Aerosp. Sci. Technol..

[8] Fu R., Wang R., Wang C., Zhang S., Wang J., Peng R., Zhu X., Kang H., Mao Y. (2025). MOFs-based aerogels and their derivatives for water treatment: A review. Environ. Res..

[9] Ren W., Wei Z., Xia X., Hong Z., Li S. (2020). CO_2_ adsorption performance of CuBTC/graphene aerogel composites. J. Nanopart. Res..

[10] Dong H., Wang T., Liu F., Luo Z., Gao X., Cai M. (2025). Unidirectional ice-templating for aerogel adsorbents: Excellent pore structure and high CO_2_ capture performance for direct air capture. Sep. Purif. Technol..

[11] Ke Q., Xiong F., Fang G., Chen J., Niu X., Pan P., Cui G., Xing H., Lu H. (2024). The Reinforced Separation of Intractable Gas Mixtures by Using Porous Adsorbents. Adv. Mater..

[12] Shin S.H.R., Jayaraman A., Thallapally P.K. (2025). Recent advances in the capture of halogen gas by porous adsorbents: A review. Sep. Purif. Technol..

[13] Wu T., Yu C., Krishna R., Qiu Z., Pan H., Zhang P., Suo X., Yang L., Cui X., Xing H. (2024). Porous materials with suitable pore size and dual-functional sites for benchmark one-step ethylene purification. AIChE J..

[14] Li J., Duan Y., Wang Y., Zhang Y., Zhou J., Zhao W., Yu J., Zhu B., Qiao K. (2024). Microenvironment modulation of interpenetrating-type hierarchical porous foam carbon by mild-homogeneous activation for H_2_ storage and CO_2_ capture under ambient pressure. J. Colloid Interface Sci..

[15] Li X., Sui Z.Y., Sun Y.N., Xiao P.W., Wang X.Y., Han B.H. (2018). Polyaniline-derived hierarchically porous nitrogen-doped carbons as gas adsorbents for carbon dioxide uptake. Microporous Mesoporous Mater..

[16] Xu Q., Lei J., Chen L., Xu J., Li G., Wan J., He L., Shen Y., Wei G., Ji G. (2024). Biomass-derived ultramicroporous carbon with narrow pore size distribution for record-high radon adsorption. Sep. Purif. Technol..

[17] Vallejos-Burgos F., de Tomas C., Corrente N.J., Urita K., Wang S., Urita C., Moriguchi I., Suarez-Martinez I., Marks N., Krohn M.H. (2023). 3D nanostructure prediction of porous carbons via gas adsorption. Carbon.

[18] Gorbounov M., Halloran P., Soltani S.M. (2024). Hydrophobic and hydrophilic functional groups and their impact on physical adsorption of CO_2_ in presence of H2O: A critical review. J. CO_2_ Util..

[19] Shilpa S., Yuan F., Li Z., Dahiya P., Mata A.C., Yadav R.M., Gao G., Hung S.F., Khan S.A., Wu J. (2025). Polymer Derived and Ni-Single Atom Doped Carbon Nanofibers for CO_2_ Capture and Electroreduction to CO. ChemSusChem.

[20] Zhu Y., Mou J., Gong X., Wang J., Feng Q., Sun X., Liu Z. (2025). Unveiling the vital role of the C-PO_3_ functional group for enhancing N_2_O adsorption on eucalyptus-based porous carbon. Fuel.

[21] Babatunde K.A., Emami-Meybodi H. (2025). Binary gas transport with multilayer adsorption in nanoporous media. Chem. Eng. J..

[22] Mo S., Wang G., Ng B.K.Y., Zhao P. (2022). Investigating porous catalysts with synchrotron X-rays and neutrons. Chem Catal..

[23] Yin Z., Chen H., Yang L., Peng C., Qin Y., Wang T., Sun W., Wang C. (2021). Investigations of CO_2_ Capture from Gas Mixtures Using Porous Liquids. Langmuir.

[24] Basok B., Davydenko B., Pavlenko A.M. (2021). Numerical Network Modeling of Heat and Moisture Transfer through Capillary-Porous Building Materials. Materials.

[25] Fraccarollo A., Canti L., Marchese L., Cossi M. (2017). Accurate Evaluation of the Dispersion Energy in the Simulation of Gas Adsorption into Porous Zeolites. J. Chem. Theory Comput..

[26] Vorokhta M., Kusdhany M.I.M., Švábová M., Nishihara M., Sasaki K., Lyth S.M. (2025). Hierarchically porous carbon foams coated with carbon nitride: Insights into adsorbents for pre-combustion and post-combustion CO_2_ separation. Sep. Purif. Technol..

[27] Shi R., Liu B., Jiang Y., Xu X., Wang H., Zeng Z., Li L. (2021). Porous carbon nanofibers with heteroatoms doped by electrospinning exhibit excellent acetone and carbon dioxide adsorption performance: The contributions of pore structure and functional groups. ACS Omega.

[28] Ghannam Z., Kamal M.S., Mahmoud M., Fawad M., Murtaza M., Ali M., Patil S. (2026). Advancing carbon dioxide mineralization for long-term carbon storage: Opportunities, challenges, and strategic recommendations. Gas Sci. Eng..

[29] Yang M., Yang B., Yue W., Zhang N., Li X., Du M., Guo L. (2024). Tunable gas absorption capability for graphene doped silica aerogel by wrinkle structure: Effects of gas absorption, mechanical properties and thermal insulation. Sep. Purif. Technol..

[30] Patil S.P., Rege A., Sagardas, Itskov M., Markert B. (2017). Mechanics of nanostructured porous silica aerogel resulting from molecular dynamics simulations. J. Phys. Chem. B.

[31] Guo L., Du M., Li J., Li W., Yang M., Xin G. (2025). Tensile Resistance and Fracture Mechanisms of Silica Aerogels Reinforced by Nanotube-Graphene Hybrid Networks. Gels.

[32] Yang M., Sheng Q., Guo L., Zhang H., Tang G. (2022). How gas-solid interaction matters in graphene-doped silica aerogels. Langmuir.

[33] Chai J., Liu S., Yang X. (2009). Molecular dynamics simulation of wetting on modified amorphous silica surface. Appl. Surf. Sci..

[34] Yang M., Guo L., Li N., Du M., Tang G. (2024). Heat treatment customizes pore structure of silica aerogel: The induced role of faults. Constr. Build. Mater..

